# Network-based method for drug target discovery at the isoform level

**DOI:** 10.1038/s41598-019-50224-x

**Published:** 2019-09-25

**Authors:** Jun Ma, Jenny Wang, Laleh Soltan Ghoraie, Xin Men, Linna Liu, Penggao Dai

**Affiliations:** 1 National Engineering Research Center for Miniaturized Detection Systems, College of Life Sciences, Northwest University, Xi’an, P.R. China; 2 Princess Margaret Cancer Centre, University Health Network, Toronto, Ontario Canada; 3 Shaanxi Microbiology Institute, Xi’an, China; 4 Department of Pharmacy, The Second Affiliated Hospital of Air Force Medical University, Xi’an, P.R. China

**Keywords:** Computational biology and bioinformatics, Target identification

## Abstract

Identification of primary targets associated with phenotypes can facilitate exploration of the underlying molecular mechanisms of compounds and optimization of the structures of promising drugs. However, the literature reports limited effort to identify the target major isoform of a single known target gene. The majority of genes generate multiple transcripts that are translated into proteins that may carry out distinct and even opposing biological functions through alternative splicing. In addition, isoform expression is dynamic and varies depending on the developmental stage and cell type. To identify target major isoforms, we integrated a breast cancer type-specific isoform coexpression network with gene perturbation signatures in the MCF7 cell line in the Connectivity Map database using the ‘shortest path’ drug target prioritization method. We used a leukemia cancer network and differential expression data for drugs in the HL-60 cell line to test the robustness of the detection algorithm for target major isoforms. We further analyzed the properties of target major isoforms for each multi-isoform gene using pharmacogenomic datasets, proteomic data and the principal isoforms defined by the APPRIS and STRING datasets. Then, we tested our predictions for the most promising target major protein isoforms of DNMT1, MGEA5 and P4HB4 based on expression data and topological features in the coexpression network. Interestingly, these isoforms are not annotated as principal isoforms in APPRIS. Lastly, we tested the affinity of the target major isoform of MGEA5 for streptozocin through in silico docking. Our findings will pave the way for more effective and targeted therapies via studies of drug targets at the isoform level.

## Introduction

Identifying the primary target associated with a phenotype can assist with exploration of the underlying molecular mechanisms of compounds and optimization of the structures of promising drugs^1^. Therefore, drug target identification is an important problem in drug discovery. Recently, a variety of computational approaches have been proposed for drug target identification, such as ligand-protein docking and network-based approaches. Traditional computational methods, such as docking, require pre-existing knowledge, including compound structures and protein sequences, and thus are often ineffective due to the limited similarity among chemical structures^2^. Network-based approaches predict novel drug target genes or drugs for repositioning through several algorithms; some of these algorithms focus on local network properties, whereas others consider the complete network topology^3–5^.

Alternative splicing (AS) is a crucial process that can generate various proteins with differential functions from eukaryoticgenes^6^. First, AS and the resulting alternative proteins are key factors in cell development and differentiation^7^. Moreover, the mechanism of drug action will be changed by interaction with alternative isoforms that have various functions at the levels of enzymatic activity, protein-protein interactions and protein-ligand docking^8^. For instance, vascular endothelial growth factor A (VEGFA) is a potent regulator of angiogenesis and capillary permeability. It plays an important role during physiological and pathological conditions. Antiangiogenic compounds generally reduce VEGFA activity for effectively inhibiting tumor growth. However, two specific VEGFA isoforms, VEGF165b and VEGF165, compete binding with bevacizumab which is used as a treatment for colorectal cancer. And therefore the VEGF165b can inhibit the effectiveness of drug bevacizumab^9^. Popel’s group^10^ showed that targeting the VEGF121 isoform was effective in reducing VEGF in tumors. Therefore, gene transcript diversity and the effect of individual protein isoforms on drug treatment results should be considered an integral part of drug design, development and therapy.

Identifying which of the alternative isoforms of a target protein is mainly related to drug effects remains a largely unsolved problem. Biological experiments investigating the effects of target isoforms after drug treatment are expensive and time-consuming. Thus, in silico methods must be developed to address this issue. RNA sequencing (RNA-seq) can accurately quantify expression data for each isoform and thus provide a useful tool for exploiting AS^11^. Previous studies have defined canonical isoforms for a given gene based on expression, topological features, transcript sequences and conservation among species^12,13^. However, we cannot simply consider the principal isoform of each gene to be the target major isoform for a drug, because isoform-level interactions are usually rewired by tissue-specific exons, and the transcript isoform of a given gene with the highest expression level is not always the longest annotated form in cell lines and tissues^14–16^. Thus, applying existing definitions and algorithms to discover the target major isoform is difficult without considering tissue-specific AS, the interactions between the drug and its target protein and drug-induced downstream changes.

**Figure 1:**
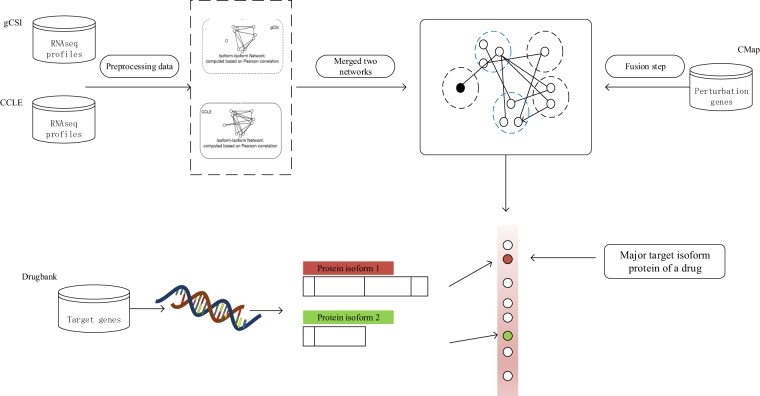
Pipeline for target major isoform prediction. Identification of the target protein isoform of a single drug. Expression profiles of common cancer cell lines in the CCLE and gCSI datasets were used separately to generate the IIC network, and connections with correlation values lower than 0.5 were removed. The coexpression values were combined using Fisher’s meta-analysis estimate algorithm to obtain the merged isoform coexpression network. The perturbation effect of a drug on a specific cell line was measured by microarray experiments in a connectivity map (CMap), and dysregulated genes were obtained from the CMap though a function in the PharmacoGx R package. The proximity score for each protein isoform was calculated as the shortest distance between each protein isoform and perturbed genes in the network. We extracted target isoforms of the target genes in the Drugbank database and found target major isoforms based on their estimated scores.

Given that the expression levels of the majority of target genes are stable after drug treatment, identifying target genes based only on gene expression data is difficult. Isik *et al*.^17^ integrated perturbed genes from drug-treated cell lines with a human protein-protein interaction network to identify drug target genes. They considered the perturbed genes to be closer to the target genes than the other proteins in the network. Inspired by this approach, we integrated isoform coexpression networks with perturbed genes to identify target genes at the isoform level. In this study, we integrated two networks generated by isoform expression data in the Genentech Cell Line Screening Initiative (gCSI)^18^ and the Cancer Cell Line Encyclopedia (CCLE)^19^ datasets to construct an isoform coexpression (IIC) network. Then, we extracted the perturbed isoforms based on functional perturbation of the corresponding gene in response to the drug and integrated these isoforms with network information to prioritize the isoforms for each known target gene (Fig. 1). We tested the accuracy of the target isoform prediction algorithm in an independent cancer IIC network and a drug-induced expression change dataset. We compared the target major isoforms with their alternative isoforms in terms of three different aspects (i.e., drug sensitivity data, known principal isoforms and proteomics data). We further validated nonprincipal isoforms that were nonetheless target major isoforms based on their expression status, functional clusters, docking tests and association between the target isoforms and drug-related biological functions. Our results indicate that understanding the major protein isoform targets of a drug is important for elucidating the mechanism of action (MoA) of that drug.

## Results

### Target major isoforms predicted by the shortest path algorithm

**Figure 2:**
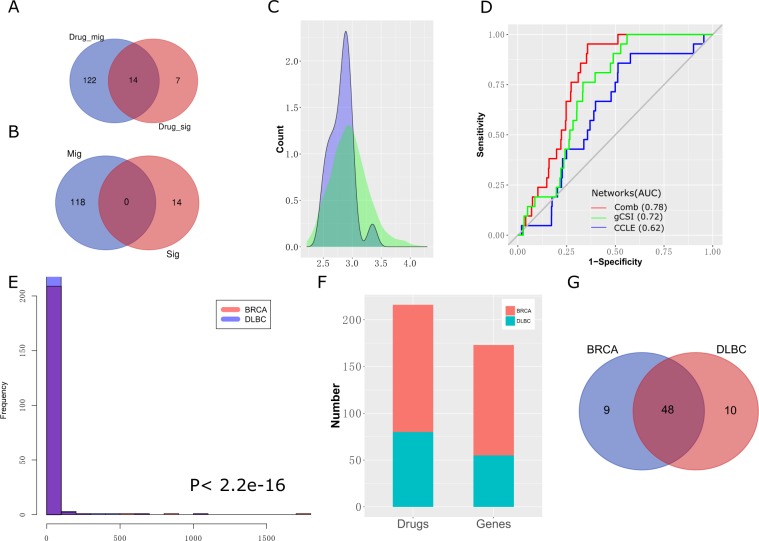
Performance test. (**A**) Numbers of drugs with MIT and SIT genes. (**B**) Numbers of MIT and SIT genes. (**C**) Distribution of the average shortest path distances of dysregulated isoforms to isoforms of known single-isoform genes (with blue) and to random (with green) targets. The two distributions are significantly different (Mann-Whitney, p-value = 0.03355). (**D**) ROC curves of three different networks, including the gCSI, CCLE and combined networks for breast cancer, with AUC values. (**E**) Distribution of cluster sizes in both the BRCA and DLBC networks. The two distributions are significantly different (Mann-Whitney, p-value < 2.2e-16). (**F**) Numbers of drugs and MIT genes in the networks. (**G**) Numbers of target major isoforms among the common target genes in the two networks.

The expression data for the isoforms in the CCLE and gCSI datasets were measured without replicate experiments. Therefore, the expression relationships are not sufficiently accurate to enable calculation using the expression profiles of the isoforms from a single dataset. We combined two isoform coexpression networks in breast cancer to generate a robust biological network^20^. Target major isoforms of 132 genes for 143 drugs were predicted by the shortest path approach (Fig. 2A,B). The average distance for the 14 isoforms of the SIT genes (mean = 2.818) in the breast cancer-based Comb network was lower than that of randomly selected isoforms (mean = 2.954). The two distributions were significantly different (Mann–Whitney, p-value = 0.03355) (Fig. 2C), indicating that the IIC network could also be used for target gene identification instead of the protein-protein interaction network. Additionally, the IIC networks built from the separate datasets (AUC_CCLE_ = 0.62, AUC_gCSI_ = 0.72) had lower AUCs than the Comb network (AUC_Comb_ = 0.78) (Fig. 2D). These results indicated that the Comb network improved the performance of the shortest path algorithm. Thus, we concluded that the Comb network was crucial for target major isoform prediction.

Due to their dynamic isoform expression profiles, the interactions of isoforms differ among various cancer cells and cancer types^8,21^. We implemented a target prediction algorithm for the leukemia-based Comb network. The topological properties of the two cancer type networks were significantly different based on their cluster size distributions (Mann-Whitney, p-value < 2.2e-16) (Fig. 2E). A total of 55 common MIT genes for 80 drugs are present in both networks (Fig. 2F). The performance of our method is quite robust for the experiment type in terms of agreement among the 48 target major isoforms (Fig. 2G).

### Association between isoforms and drug sensitivity data

**Figure 3:**
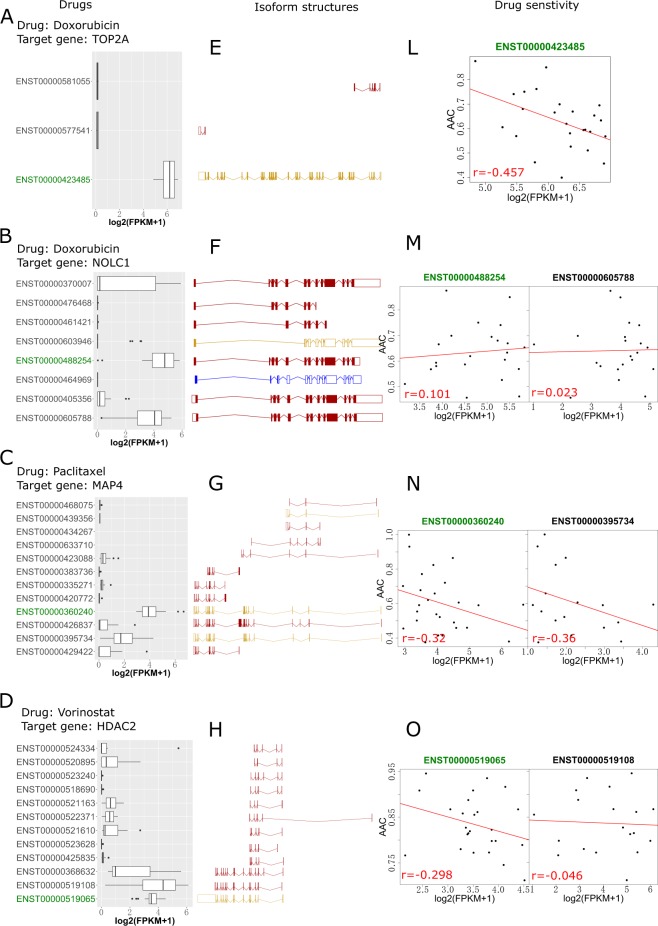
Association between the isoform expression levels of target genes and drug responses. Panels A–D show the isoform expression levels of the target genes (in the network) for drugs used in the gCSI and CMap datasets, including vorinostat, paclitaxel and doxorubicin. Panels E–H visualize the structure of each transcript isoform using the Ensembl Genome Brower. Red indicates transcripts that are protein-coding isoforms in the Ensembl database. Protein-coding isoforms annotated by Ensembl and Havana (shown in yellow and blue, respectively) represent processed transcripts. Panels L–O show the associations between isoform expression and the drug sensitivity data (AAC).

Given recent concerns about pharmacogenomic data obtained using cell lines, such as those available in gCSI, we compared the association between target isoforms and drug sensitivity data. Doxorubicin, paclitaxel and vorinostat are common drugs that are used in both the gCSI and CMap datasets. The target major isoform of each gene is strongly associated with the drug response (Fig. 3L–O) and is highly expressed in 47 overlapping breast cancer cell lines in the CCLE and gCSI datasets (Fig. 3A–D), indicating that the expression values of the target major isoforms are closely related to the drug response. For example, NOLC1 produces eight protein isoforms, although only two isoforms are highly expressed in breast cancer cell lines (Fig. 3B). The target major isoform of NOLC1 has a stronger relationship with the drug response than the isoform with lower expression (ENST00000605788) (Fig. 3M). Additionally, the length of the isoforms was not correlated with either their expression status in specific cancer cell lines or with drug effects (Fig. 3E–H). For instance, ENST00000519065 expressed by HDAC2 has a longest sequence and is closely related to the drug response but has a lower expression value comparing with ENST00000519108. (Fig. 3D,H). Although the target major isoform of MAP4 has a similar extent of isoform-drug response association and length as the other isoforms, it is highly expressed among breast cancer cell lines (Fig. 3C,G,N). All of the results indicated that the target major isoforms were closely associated with drug sensitivity. The complex elements of the target isoforms, such as the expression value, length and number of target genes per drug, contribute to the drug response.

### Comparison of the target major isoforms, longest isoforms and most conserved isoforms

Canonical isoforms in different databases have been defined using various methods based on conservation, expression data, length or the number of connected isoforms^12,22–24^. The STRING database, which provides human protein interaction data, usually chooses the longest isoform of MIT genes^23^. The principal isoform in the APPRIS database is defined by merging protein structural information, functionally important amino acid residues and cross-species conservation information^12^. We detected the target major isoforms based on the three properties of length, conservation and translation of the isoform. We identified the target major isoform for a total of 118 MIT genes and calculated the proportion of target major isoforms with isoforms annotated in the APPRIS and STRING datasets (Table 1). The proportions of overlapping MIT genes based on the two isoform types were 82.2% for the principal isoforms and 63.5% for the longest isoforms (P = 0.001). Then, we found that 58 MIT genes had isoform expression evidence at the protein level and that the target major isoforms of 44 multi-isoform gene targets were translated to proteins in breast cancer cell lines. The overlap was statistically significant (P = 0.001) compared with the number of alternative isoforms that overlapped expressed protein isoforms by chance (29 ± 3). Based on the above comparison results, most target major isoforms are the principal and longest isoforms of a single gene and are highly translated proteins.

**Table 1:** Comparison of a number of target major isoforms in terms of three properties.

	Target genes	Shared target genes (%, P-value via random permutation)	Chance
APPRIS principal isoforms	118	97 (82.2%, P = 0.001)	41 ± 4 (34.7% ± 3%)
STRING longest isoforms	118	75 (63.5%, P = 0.001)	26 ± 4 (22% ± 3%)
Proteomic isoforms	58	44 (75.8%, P = 0.001)	29 ± 3 (50% ± 5%)

### Drugs with non-APPRIS target major isoforms

To further elucidate the difference between the nonprincipal target major isoforms and the alternative isoforms of one MIT gene, we grouped the target major isoforms into APPRIS matched and non-APPRIS matched groups based on the annotated isoforms in the APPRIS dataset. Then, we separately selected the top 5 target isoforms ranked by the shortest distance score from the two groups for further study.

**Figure 4:**
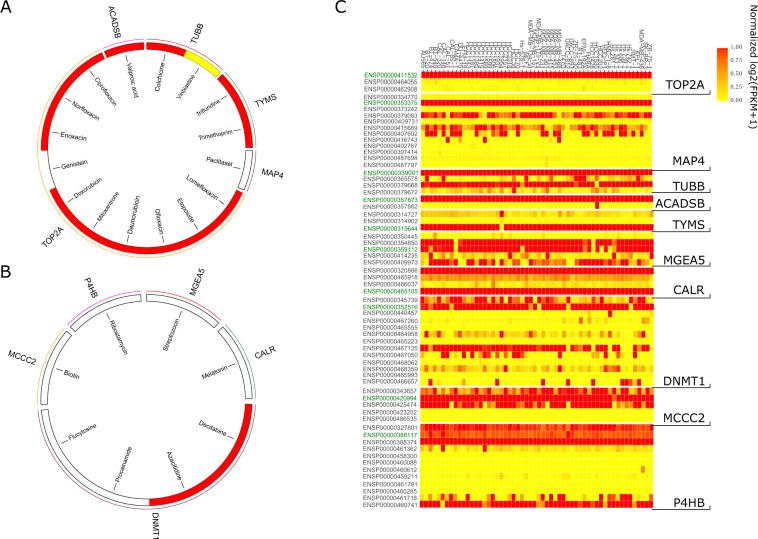
Drug action and expression profiles of the target major isoforms in the two groups. (**A**) The responses of target genes to drugs in the APPRIS group. (**B**) The responses of target genes to drugs in the non-APPRIS group. Red indicates that the drug is an inhibitor of the given target, yellow indicates an adduct and white denotes an unknown effect. (**C**) Expression of selected isoforms among 47 breast cancer cell lines. Log_2_(FPKM + 1) of each isoform was normalized based on the corresponding gene.

Ligands are defined as antagonists, inducers or inhibitors based on the effects of the compounds on their target proteins. For example, doxorubicin, which is an inhibitor of DNA topoisomerase 2-alpha (TOP2A), exhibits anticancer effects by inhibiting TOP2A activity and suppressing DNA synthesis^25^. Circos plots illustrate the action of the drug on the target gene, whose corresponding target major isoforms are categorized as APPRIS and non-APPRIS (Fig. 4A,B). In contrast to the non-APPRIS group, the interactions between target genes and drugs in the APPRIS group are suggested by the literature in the Drugbank database, which is a richly annotated database that contains detailed drug data, such as drug targets and drug actions^26^. Figure 4C shows expression data for the isoforms for each target gene among 47 breast cancer cell lines. For genes in the APPRIS group, three selected genes with known drug actions generate a single highly expressed isoform. Conversely, more than one highly expressed isoform for each gene exists in the non-APPRIS group. Thus, these results indicated that the first problem in exploring the complex mechanisms of drug activities is identifying which isoform is the target major isoform for a gene that generates multiple highly expressed isoforms.

**Figure 5:**
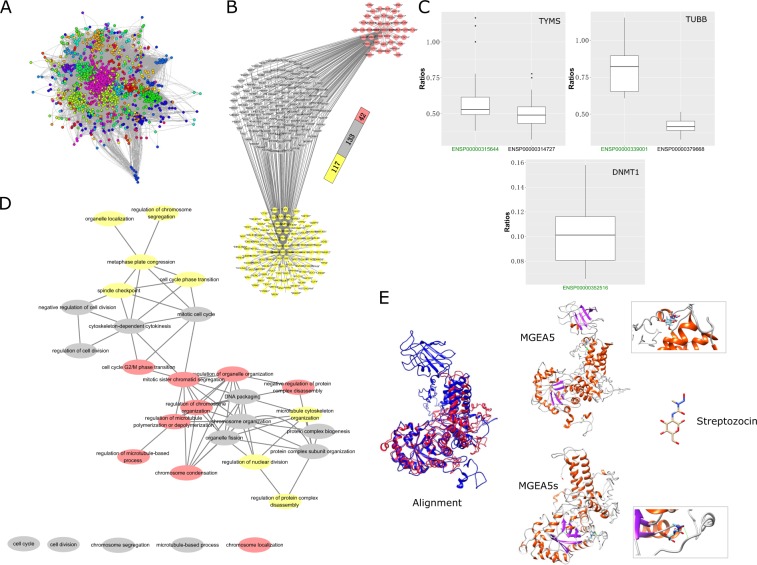
Non-APPRIS annotated target major isoforms. (**A**) Clusters in the breast cancer type-based isoform coexpression network. (**B**) All connected nodes of isoforms of the target gene TYMS. Gray indicates common neighbors, yellow denotes the specific neighbors of the target major isoform and red represents the specific neighbors of the alternative isoform. (**C**) Ratio of the number of significant neighboring isoforms of the genes TYMS, TUBB and DNMT1 with the genes perturbed by the drugs trifluridine, colchicine and azacitidine in each common gene set. The ratio distributions for isoforms of TYMS and TUBB are significantly different (Mann-Whitney, p–value = 0.009026 and 0.0001554). (**D**) Overlapping biological functions between the target isoforms of TYMS and drug perturbation genes. The connections among each biological process were generated by the REVIGO website^54^ and illustrated by Cytoscape. (**E**) Structural differences between two isoforms and their affinity activity.

We investigated the cluster properties of the cancer-based IIC network to explore the biological processes of each potential target isoform (Fig. 5A). Figure 2E shows the distribution of the cluster size of the breast cancer-based isoform coexpression network, which includes 217 clusters. The number of small clusters (with a size <10) was larger than the number of large clusters (191 vs. 26). Compared with their alternative isoforms, the target major isoforms of tubulin beta chain (TUBB), DNA (cytosine-5)-methyltransferase 1 (DNMT1), MGEA5 and protein disulfide-isomerase (P4HB) in either the APPRIS or the non-APPRIS group are strongly associated with larger clusters and are mostly related to the number of biological processes (Table 2). We speculated that the target major isoforms of each gene played crucial roles in cell development. Meanwhile, the isoforms of thymidylate synthase (TYMS), calreticulin (CALR) and methylcrotonoyl-CoA carboxylase beta chain (MCCC2) were separately involved in the same clusters, indicating that using cluster analysis to interpret the functions of these isoforms is difficult. Additionally, the member isoforms of large cluster 59, which included CALR and MCCC2, were not significantly enriched in any biological processes. The reason for this result is that the random Walktrap algorithm identifies clusters based on their topological features in the network, which may assign nodes with diverse biological pathways to the same cluster^27^.

**Table 2:** Target major isoforms are related to diverse functional processes.

Types	Target genes	Isoforms	Clusters (Size)	No. biological process
APPRIS	TUBB	ENSP00000339001^*^	63(608)	138
		ENSP00000379668	4(558)	84
	TYMS	ENSP00000315644^*^	63(608)	138
		ENSP00000314727	63(608)	138
Non APPRIS	DNMT1	ENSP00000352516^*^	7(1740)	127
		ENSP00000345739	10(7)	0
	MGEA5	ENSP00000359112^*^	7(1740)	127
		ENSP00000354850	3(119)	5
	CALR	ENSP00000465105^*^	59(876)	0
		ENSP00000320866		
		ENSP00000465918		
	MCCC2	ENSP00000420994^*^	59(876)	0
		ENSP00000343657		
	P4HB	ENSP00000388117^*^	7(1740)	127
		ENSP00000327801	2(1079)	69
		ENSP00000460741	67(4)	0

^*^Denotes the target major isoforms. The reason for choosing TUBB and TYMS from the APPRIS group is that the isoforms of these genes are well known, and more than one isoform appeared in the networks.

The CMap provides a useful tool for screening associations between compounds and identifying highly correlated gene expression patterns. These results have the potential to identify novel pathways or genes involved in a complex biological function^28^. To further identify target major isoforms within the same cluster, we independently extracted connected isoforms of the target isoforms (Fig. 5B, Supplementary Table S1). We also obtained 470 perturbed genes for trifluridine (target gene TYMS), 510 perturbed genes for colchicine (TUBB) and 2,998 perturbed genes for azacytidine (DNMT1) from the CMap database. Then, we performed GO term enrichment analysis of the direct neighbors of the isoforms and the perturbed genes of each drug. The overlapping gene sets between the neighbors and the perturbed genes were used to calculate the proportion of the number of neighbors with perturbed genes within each common gene set (Supplementary Table S2). Figure 5C shows the distribution of ratios between isoforms for each target gene. The ratios are significantly different for the target major isoforms of TYMS and TUBB (Mann-Whitney, p-values = 0.009026 and 0.0001554, respectively), indicating that the target major isoforms play a more important role than the alternative isoforms of the same gene for understanding the mode of action of a drug. Additionally, 13 of 30 overlapping gene sets were shared among the target isoforms (Fig. 5D). These results indicate that the isoforms of each target gene exhibit similar or variant patterns that are correlated with the modes of action of the drugs.

**Figure 6:**
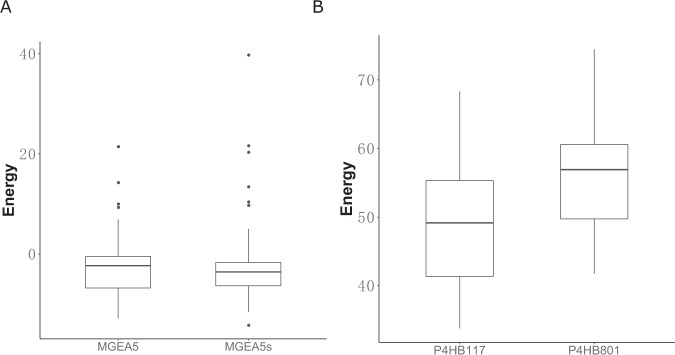
Distribution of energy of binding modes between drug and isoforms. (**A**) Distribution of binding modes energy between streptozocin and MGEA5 is similar with MGEA5s modes (Mann-Whitney, p–value = 0.505). (**B**) ENSP00000388117 (P4HB117) had a significant lower interaction energy binding with ribostamycin comparing with ENSP00000327801 (P4HB801) (Mann-Whitney, p–value = 0.000872).

Differences in the protein sequences of the two isoforms lead to the production of different 3D structures, which impact the interaction between ligands and proteins. MGEA5s is a splice variant without a putative acetyltransferase domain at the C-terminal end of MGEA5^29^ (Fig. 5E). The interaction energies for the docked complexes were calculated by SwissDock and summarized in Supplementary Tables S3 and S4. The algorithm of SwissDock includes several steps: First, generation of binding modes (BMs). Secondly, the energies of each BM are calculated by Chemistry at HARvard Molecular Mechanics (CHARMM) program^30^. Then, clustered and ranked BMs with the most favorable energies based on the solvent effect. Lastly, the favorable clusters of BMs are output into the result file. We compared the interaction between the target major isoform of a protein (ENSP00000359112, known as MGEA5s) and streptozocin, which is an antibiotic that is produced by *Streptomyces achromogenes*, with the interaction between the principal isoform (ENSP00000354850, known as MGEA5) and the same drug. There are 46 BMs for MGEA5 and 48 BMs for MGEA5s. Table S3 shows the most favorable energies of each BM across MGEA5 isoforms. We found that the energy distribution of MGEA5s’s binding modes are similar with MGEA5 (Mann-Whitney, p–value = 0.505). Ribostamycin, also antibiotic, was reported as P4HB inhibitor that suppresses the chaperone-like activity^31^. 49 BMs of P4HB117-ligand complexity was identified while 45 predicted BMs for P4HB801 binding complexity. Interesting, the less length of ENSP00000388117 of gene P4HB (P4HB117) has less interactive energy of P4HB117-ligand complexity based on simulation results comparing with ENSP00000327801 (P4HB801) (Figs 6 and S2). Therefore, all results indicate that target main isoforms can efficiently binding with compounds.

## Discussion

The half-maximal effective concentration (EC50) or half-maximal inhibitory concentration (IC50), inhibition constant (Ki) and dissociation constant (Kd) were measured by biological experiments to identify the drug target. However, the *in vitro* or *in vivo* assays are time-consuming and costly to determine all possible drug targets. Molecular docking-based methods are widely used traditional approaches rely on the 3D structures of targets^32^. The scoring function of molecular docking-based methods evaluate drug targets by calculating the docking scores correlated with binding affinities. Therefore, molecular docking-based methods are often limited by poor-quality 3D structures. As systems biology and network pharmacology are rapidly developing, several computational approaches have provided valuable strategies for the systematic prediction of potential drug targets^33^. Compared to the molecular docking-based methods, the network-based methods are simple, fast and independence from the 3D structures of drug targets. Network-based methods predict promising drug targets by performing simple processes such as diffusion or random walk on networks^4,17^. These processes can be considered as matrix multiplication mathematically. Genes produce multiple isoforms with diverse functions due to alternative splicing processes. Drugs usually bind target proteins and then influence downstream processes. Therefore, drug target identification at the isoform level is also crucial for understanding the modes of action of drugs, which is more consistent with those observed in reality. Biological networks, such as protein-protein interaction and coexpression networks, provide valuable methods for exploring system-level properties^34^. Our study is the first to identify target major isoforms for each MIT gene by integrating network features with drug-induced transcriptional responses. We observed that the merged IIC network improved the performance of the shortest path algorithm and that the majority of the target major isoforms of MIT genes for a specific drug were stable and barely affected by the tissue type. Furthermore, target major isoforms are highly expressed and are more strongly associated with the drug response than their alternative isoforms. Target major isoforms overlap significantly with principal isoforms, as defined by several properties, and are highly expressed at the protein level. Importantly, we compared the target major isoforms and the principal isoforms of different genes at four levels, including expression data, topological features (such as clusters and hubs), the biological pathways of the drug and ligand and protein docking, to validate nonprincipal target isoforms. Because the drug targets were resolved at the protein level, we did not need to consider isoforms with untranslated regions. We reduced the computation time by using only the protein-coding isoforms from Ensembl mRNA data in the expression calculation.

The gene expression profiles of cells will change depending on the tissue type or growth period. Thus, the topological properties of gene/isoform coexpression networks and drug-induced differential expression data are cancer type-specific. Our hypotheses are supported by the high consistency between the leukemia and breast cancer datasets at the level of the target major isoforms. Most drugs with the same target genes share a single target major isoform in the context of different cancer types, although a drug with cancer-specific target isoforms may have different modes of action in a given cancer. For example, trifluridine’s target gene TYMS produces two isoforms (ENSP00000314727 and ENSP00000315644). ENSP00000315644 was predicted as a target major isoform using a breast cancer-based IIC network, whereas ENSP00000314727 was identified by a leukemia-based IIC network.

Polypharmacology focuses on understanding drugs that interact effectively with multiple targets. Several lines of evidence suggest that many effective drugs achieve their effects through multiple rather than single targets^4,35^. On the one hand, some drug targets seem to be closely related to drug reactions. On the other hand, some targets may have less correlation with drug responses and may even lead to unexpected side effects. For example, doxorubicin has two target proteins (TOP2A and NOLC1). In this study, the target major isoform (ENST00000423485) of TOP2A had a stronger correlation with drug sensitivity than the target major isoform (ENST00000488245) of NOLC1 for the same drug. Given that drugs with a common target usually have the same target major isoform, the identification of the target major isoform for each gene requires additional genomic profiles of the effects of these drugs to reduce the impact of multiple targets on the drug response.

Different databases have used multiple lines of evidence to find the principal isoforms of multiple-isoform genes. To date, there are no standard criteria to define principal isoforms. Previous definitions of principal isoforms have focused on individual isoforms and have not been applied at the systems level or in the context of tissue type^24^. Meanwhile, compounds not only act on principal isoforms but also bind other highly expressed isoforms of the same target gene, thereby complicating the drug’s mode of action. Our results indicated that principal isoforms should not be considered adequate evidence to identify target major isoforms.

Our statistical modeling incorporates biological networks and drug-correlated transcriptional data to approach the true association of target isoforms with a given drug. However, we should note the limitations of this method. First, for the more than 1,000 drugs in the CMap dataset, this method could identify target major isoforms for only 118 MIT genes and 136 drugs. The reason for the limited prediction capability of the method was that most target isoforms were removed to obtain robust connections among the isoforms in the Comb IIC network. A larger pharmacogenomic dataset with reliable transcriptome data or a more cancer type-specific network will be necessary to overcome this limitation. A second limitation lies in the lack of isoform perturbation data, because all published pharmacogenomic datasets are at the gene level. To address this challenge, we consider all alternative isoforms of perturbed genes as perturbed isoforms.

Further validation experiments would help further increase the impact of the work, and strengthen the association between compounds and target principal protein per gene in the context of cancer types. Saccharomyces cerevisiae expression system is an ideal system to test the affinity ability between target isoform proteins and drug target. The functional experiments, such as RNAi approach is also needed to elucidate the correlation between compounds and target isoform protein.

In summary, this study integrates gene expression profiles with cancer type-specific IIC networks to prioritize the target isoforms of well-known drug targets. Compared with the alternative isoforms of the same gene, target major isoforms are dependent on the cancer type, are highly expressed *in vitro* and are strongly associated with the drug response. We found that the nonprincipal isoforms of DNMT1, MGEA5 and P4HB4 were the target major isoforms for azacytidine, decitabine, procainamide, flucytosine, streptozocin and ribostamycin based on various properties. Although our results provide important insights into drug targeting at the isoform level, more studies are required to examine the role of target major isoforms in cancer progression, treatment and personalized therapy.

## Methods

### Building isoform coexpression networks

We created a coexpression network at the isoform level through the following steps introduced in our previous publications^24,36^. First, expression data for isoforms from overlapping cell lines of the same cancer type in the CCLE and gCSI datasets were downloaded from the PharmacoGx platform (version 1.12.0)^37^, which comprises pharmacological profiles for several hundred cell lines. The updated CCLE and gCSI PharmacoSets contain isoform-level expression data processed from raw RNA-seq profiles extracted from CGHub^38^ and NCBI GEO^39^. Zhaleh *et al*.^40^ aligned the RNA-seq reads to the Ensembl Genome Reference Consortium release GRCh38^41^ using HISAT2^42^, annotated the isoforms and calculated their expression with StringTie^43^. A total of 58,037 genes, including 19,950 protein-coding genes, 15,767 long noncoding RNAs (lncRNAs) and 14,650 pseudogenes, was annotated by Gencode (version 25)^44^. Then, the FPKM values (the number of fragments per kilobase per million mapped reads units) were converted to log2 (FPKM + 1) to obtain the expression values of the isoforms. Noncoding isoforms were removed based on Ensembl identifiers using the R package BiomaRt (version 2.34.3)^45^. We calculated the Pearson correlation coefficients of two isoform expression values for each dataset as follows:

$${\rho }_{ij}=\frac{cov({E}_{i},{E}_{j})}{{\sigma }_{{E}_{i}}{\sigma }_{{E}_{j}}}$$

where E is the expression value of protein isoforms i and j. The value log2 (FPKM + 1) was ≥1 in at least 30 cancer cell line types in each dataset. Protein isoforms i and j are also common isoforms in both the gCSI and CCLE datasets.

Interactions between isoforms in the same genes were removed. To find a balance between removing weak interactions and keeping more isoforms in the network, the isoform network was filtered by the threshold s = 0.5, which was calculated as follows:

$${N}_{ij}=\{\begin{array}{cc}{\rho }_{ij} & |{\rho }_{ij}| \ge 0.5\\ 0 & {otherwise}\end{array}$$

### Combined networks and their topological characteristics

To address the lack of reproducibility of RNA-seq measurements across studies^18,19^, we applied a meta-analytical approach to combine the two isoform networks. First, we tested the stability of the networks through the Pearson correlation coefficients (Cor) of each isoform degree in the two networks (Fig. S1). Then, the two networks of one cancer type with Cor > 0.5 were merged by the combine.est function in the survcomp (version 1.28.5) R package after a z transform for the coefficient values of each network^20^. The combined isoform coexpression (Comb) network for breast cancer included 6,250 isoforms and 294,098 stronger edges. The leukemia-based Comb network contained 4,670 isoforms and 107,432 connections.

A Walktrap approach is a hierarchical structure algorithm proposed by Pons, which assumes that short random walks tend to stay in the same cluster^46^. We identified clusters of combined networks using the Walktrap (CW) function in the igraph package (version 1.2.1) with the default parameters^47^. A total of 26 of the 217 clusters in the breast cancer type-based combined network contained more than 10 members. In contrast, the leukemia-based combined network generated 751 clusters, including 730 smaller cluster (size < 10).

### Dysregulation of gene expression of drug in the connectivity map (CMap) and its target gene

We preprocessed the CMap data using the drug perturbation signature function of the PharmacoGx package^37^. The details of this function were described on our pervious publications^24^. We created a signature for each drug by fitting a linear regression model to the effect of the drug concentration on gene expression in cell lines and adding a term to control for the batch effect in the CMap dataset:

$${\rm{G}}={\beta }_{0}+{\beta }_{i}{C}_{i}+{\beta }_{t}T+{\beta }_{d}D+{\beta }_{b}B$$

where *G* stands for molecular feature expression (Gene), *Ci* indicates the concentration of a given compound, *T* denotes the cell line type, *D* represents the duration of the experiment and *B* represents the regression coefficient. The significance of the association between a drug and genes was estimated by *β*_*i*_, which was calculated using an F-test to determine the improvement in fit after inclusion of the term. Genes with a P-value < 0.01 after preprocessing were considered dysregulated, and their absolute t-statistic value was used as differential expression data.

The target genes of drugs used for treatment in the CMap database were downloaded from Drugbank (www.drugbank.ca/releases/5-0-11/downloads/target-all-uniprot-links)^48^, and the gene symbols were obtained by matching the UniProt identifiers of target genes in the drug target identifier file (www.drugbank.ca/releases/5-0-11/downloads/target-all-polypeptide-ids). We retained 132 target genes that were present in the breast cancer-based combined network for further study. We divided the target genes into single-isoform target (SIT) genes and multi-isoform target (MIT) genes based on the number of isoforms per gene in the Ensembl database^41^. SIT genes of the selected targets were used to evaluate the performance of the drug target prediction approach.

### Scoring systems

In most studies, alteration of expression profiles is recorded at the gene level. To solve this problem, we converted the differentially expressed genes to their corresponding isoforms based on the Ensembl database using the BiomaRt R package. We expressed the formula used to calculate the shortest path score of n in network N as follows:

$${\rm{S}}=\sum _{Pr\in DI}sp(n,Pr,N),n\in N$$

where *Pr* represents the isoform perturbed by the drug and *DI* indicates the total number of dysregulated isoforms. Lastly, we sorted the isoforms by their scores in increasing order. We randomly chose 1,000 different nontarget isoforms to calculate the shortest path distance of dysregulated genes to random isoforms.

### Performance evaluation

Given the lack of curated drug targets at the isoform level, we used SIT genes to assess the prediction performance of the LR approach using the receiver operating characteristic (ROC) curve as described in the study by Laenen *et al*.^4^ to define TP (true positive), FN (false negative), FP (false positive) and TN (true negative) predictions. The true positive rate (TPR) and false positive rate (FPR) were calculated at all possible thresholds in each network type, such as from 1 to 10,937 in the CCLE network and from 1 to 6,263 in the combined network, for the ranked list of drug target isoforms. the predictions were divided into true and negative sets depending on each cutoff. The TPs were all correctly predicted known targets above or equal to the rank cutoff. The FPs were all proteins ranked above the cutoff that were not in the known target set. The FNs were known drug targets that were ranked below the cutoff. All remaining proteins were defined as TNs. We constructed the ROC curve for the TPR and FPR of the different rank cutoffs and finally calculated the area under the ROC curve.

### Pre-existing definitions for major isoforms per gene

We downloaded the 19,247 longest isoforms of *Homo sapiens* from the STRING database (9606.protein.links.v10.txt.STRING download; available at https://stringdb.org/cgi/download.pl?UserId=6µqaFS2HsxDM&sessionId=X14NRwQRfV6D&species_text=Homo+sapiens) and 34,817 principal isoforms of the Gencode27/Ensembl90 version from the APPRIS website (appris_principal.cvs [APPRIS Downloads; available at http://appris.bioinfo.cnio.es/#/ downloads; accessed 8/Dec 2017]) for comparison with the MIT gene isoforms. Within the MIT genes, we removed those that could not be mapped into these datasets before the comparison study. To calculate the P-value for the percentage of target major isoforms for each comparison, we performed a 1,000-fold permutation test by randomly selecting the same number of isoforms from each MIT gene.

### Proteomic data from three breast cancer cell lines

We also tested the agreement between the main splice variant proteins and principal target isoforms. Splice variant proteins were generated based on our published identification method^24^. The steps are as follows: firstly, mass spectrometric results data of different breast cancer subtypes was download from the PRIDE archive (PRIDE archive download; available at http://proteomecentral.proteomexchange.org/cgi/GetDataset?ID=PXD006703)^49^. Secondly, the Uniprot IDs marked with “Majority protein IDs” were extracted from these files and retained for the further study. Lastly, we obtained Ensembl IDs of these variant proteins via mapping the UniProt IDs on the UniProt website. A total of 2,074 genes with main variant proteins were obtained from the archive’s mass spectrometric search result files, which included 58 MIT target genes for 73 CMap drugs. We performed the same procedure to compute the P-values of the comparisons.

### Isoform sensitivity identification for vorinostat, paclitaxel and doxorubicin in breast cancer

A previous study developed a pipeline to process raw pharmacological data from CCLE and gCSI and to generate drug dose-response curves using standard curve fitting algorithms^37^. The area under the curve (AUC) values were computed by integrating all drug dose-response data points to summarize the drug response. Only three drugs (vorinostat, paclitaxel and doxorubicin) were used in both the gCSI and CMap datasets. We obtained the AUC values of these drugs in gCSI using the PharmacoGx platform and used the drug dose-response curve (AAC = 1 − AUC) to evaluate drug sensitivity. To figure out the association between isoform expression data and drug sensitivity, we compute pearson correlation coefficients for the target isoforms of each drug in the overlap breast cancer subtype cell in the CCLE and gCSI datasets.

### Affinity of a drug for the target isoforms

The PDB structures of the drug streptozocin and ribostamycin were downloaded from Drugbank (www.drugbank.ca/). The protein sequences of two MGEA5 isoforms (ENSP00000359112, known as MGEA5s, and ENSP00000354850, known as the principal isoform) were obtained from the UniProt database, and the tertiary structure of each MGEA5 isoform was predicted by the I-TASSER server^50^, which is a platform for automated structure prediction tools. We compared the two protein structures of MGEA5 using TM-align^51^. SwissDock was used to detect the binding modes between streptozocin and the MGEA5 isoforms^32^. Each mode was scored based on its FullFitness and clustered. All structure-related features were visualized using UCSF Chimera^52^. We used the same pipeline for comparing the 3D structures of P4HB’s isoforms and the affinity ability with ribostamycin.

### Functional enrichment analysis

The biological processes in Gene Ontology (GO), which provide gene functions and gene products in 3 categories [biological process (BP), molecular function (MF) and cellular component (CC)], were downloaded from the Molecular Signatures Database (http://software.broadinstitute.org/gsea/msigdb/index.jsp). We enriched connected isoforms of each predictor isoform, members of each cluster and perturbed genes into biological process GO terms to annotate their function with a hypergeometric test using the Piano R package (version 1.18.1)^53^. Biological process GO terms with a false discovery rate (FDR) < 0.05 were further considered.

## Supplementary information

Supporting Information

## Data Availability

The pharmacogenomics data used in this study are publicly available through PharmacoGx platform. CCLE is available from https://portals.broadinstitute.org/ccle/. The gCSI dataset is available from the European Genome-phenome Archive (EGAS00001000610).
