# Supplementary material for: Network-based method for drug target discovery at the isoform level
Source: Sci Rep. 2019 Sep 25;9:13868. doi: 10.1038/s41598-019-50224-x (PMC6761107; doi:10.1038/s41598-019-50224-x)
Supplement: Supplementary file 1 — Supporting Information [file 41598_2019_50224_MOESM1_ESM.pdf]

Network-based method for drug target discovery at the isoform level

Jun Ma[1,2], Jenny Wang[2], Laleh Soltan Ghoraie[2], Xin Men[3], Linna Liu[4*] and Penggao Dai[1*]

[1] National Engineering Research Center for Miniaturized Detection Systems, College of Life Sciences, Northwest University, Xi'an, PR China
[2] Princess Margaret Cancer Centre, University Health Network, Toronto, Ontario, Canada
[3] Shaanxi Microbiology Institute, Xi'an, China
[4] Department of Pharmacy, The Second Affiliated Hospital of Air Force Medical University, Xi'an, PR China

* Correspondence:

Penggao Dai daipg@nwu.edu.cn
Linna Liu liulinna@fmmu.edu.cn

Table S1. Number of directly neighbors of target isoforms

Target genes	Major isoforms	Alternative isoforms	Common isoforms
TYMS	117	42	133
TUBB	265	106	20
DNMT1	102	8	0

Table S2. The overlapping gene sets between the neighbors of target isoform and the perturbed genes of azacytidine and colchicine.

Drug/Target isoform	Go ID	Go term	Significant (in gene set).x	
			Neighbors genes	Perturbed genes
Azacytidine / ENSP00000352516 (DNMT1)	GO:0000070	MITOTICSISTERCHROMATIDSEGREGATION	6	38
	GO:0000278	MITOTICCELLCYCLE	20	250
	GO:0000375	RNASPLICINGVIATRANSESTERIFICATIONREACTIONS	9	89
	GO:0000819	SISTERCHROMATIDSEGREGATION	8	67
	GO:0006396	RNAPROCESSING	17	232
	GO:0007049	CELLCYCLE	25	381
	GO:0007059	CHROMOSOMESEGREGATION	9	89
	GO:0008380	RNASPLICING	11	115
	GO:0010639	NEGATIVEREGULATIONOFORGANELLEORGANIZATION	10	124
	GO:0022402	CELLCYCLEPROCESS	22	329
	GO:0031110	REGULATIONOFMICROTUBULEPOLYMERIZATIONORDEPOLYMERIZATION	8	57
	GO:0032886	REGULATIONOFMICROTUBULEBASEDPROCESS	9	72
	GO:0044770	CELLCYCLEPHASETRANSITION	9	88
	GO:0048285	ORGANELLEFISSION	13	137
	GO:0051301	CELLDIVISION	12	144

	GO:0098813	NUCLEARCHROMOSOMESEGREGATION	8	72
	GO:0140014	MITOTICNUCLEARDIVISION	13	112
Colchicine/ ENSP00000379668 (TUBB)	GO:0000278	MITOTICCELLCYCLE	19	46
	GO:0007049	CELLCYCLE	23	70
	GO:0008104	PROTEINLOCALIZATION	33	82
	GO:0022402	CELLCYCLEPROCESS	21	59
	GO:0033365	PROTEINLOCALIZATIONTOORGANELLE	17	33
	GO:0051649	ESTABLISHMENTOFLOCALIZATIONINCELL	34	81
	GO:0061024	MEMBRANEORGANIZATION	22	49
	GO:0070727	CELLULARMACROMOLECULELOCALIZATION	28	60

Table S3. Interaction energies of binding modes between MGEA5 isoforms and streptozocin. The top 1 of the most favorable energies of each cluster

MGEA5		MGEA5s	
Cluster	Energy	Cluster	Energy
6	-12.8924	0	-14.266
0	-12.8007	3	-11.5858
9	-12.7308	2	-10.8692
4	-12.4779	5	-10.2236
1	-9.81554	7	-10.018
27	-9.07826	17	-9.09553
22	-8.70199	1	-8.10268
32	-8.49119	27	-7.8128
16	-8.46287	33	-7.79599
37	-8.30152	4	-7.72244
3	-7.91542	6	-6.93462
12	-7.24422	10	-6.41296
5	-5.43518	8	-6.32839
2	-5.32669	14	-6.1091
19	-4.87679	26	-6.02083
21	-4.85919	18	-5.99564
28	-4.76573	13	-5.83311
40	-4.56697	20	-5.58181

38	-4.05524	9	-5.24733
20	-3.37884	16	-4.99279
18	-2.80597	35	-4.72542
7	-2.67559	21	-4.1728
23	-2.55419	24	-3.64189
11	-2.11119	28	-3.63547
31	-1.99424	15	-3.56357
25	-1.90592	12	-3.11121
29	-1.71188	29	-2.96831
15	-1.70083	25	-2.96008
8	-1.67144	34	-2.66987
13	-1.4255	22	-2.57935
10	-0.82486	32	-2.2769
24	-0.8009	11	-2.2549
35	-0.74283	38	-2.10621
36	-0.70357	31	-2.06577
30	-0.41894	23	-2.00601
33	-0.36663	19	-1.72532
14	-0.18447	30	-1.61898
17	0.183428	36	-0.89034
26	0.493536	37	1.64609
34	1.0949	40	2.19868
42	6.61025	39	3.56013
39	6.89255	41	5.00508
41	9.2822	42	9.67997
43	9.96118	43	10.3729
44	14.2395	44	13.4119
45	21.4244	45	20.2971
		46	21.6084
		47	39.7369

Table S4. Interaction energies of binding modes between P4HB isoforms and ribostamycin. The top 1 of the most favorable energies of each cluster

P4HB801		P4HB117	
Cluster	Energy	Cluster	Energy
8	41.7279	13	33.7715
3	41.9289	1	34.7747
0	42.9428	26	35.9546
18	43.424	8	38.0262
29	43.9528	7	38.4036
15	44.5011	16	38.9935
20	46.1096	25	39.0297
5	47.0715	15	39.0976
1	47.5654	3	39.6965

14	47.8474	38	40.0764
35	49.5979	6	40.6389
28	49.7473	12	40.691
24	50.5266	4	41.3368
17	51.0663	14	41.7459
10	51.4667	2	44.1348
37	52.6126	0	44.5528
11	53.1807	34	44.5754
6	53.6303	24	45.352
2	53.8073	36	46.1525
13	55.0218	18	46.3995
7	55.3076	41	46.7367
21	55.3386	10	47.1245
16	56.9098	22	48.0463
26	56.9118	40	48.0802
23	57.3313	28	49.1543
27	57.3462	35	49.3357
33	57.8576	17	50.0439
19	58.1125	27	50.2874
4	58.2854	31	50.4186
25	58.3175	45	50.6819
32	58.9879	32	50.7196
9	59.607	21	50.7291
39	60.2951	9	51.4086
36	60.5855	5	51.7445
31	60.784	33	53.3438
12	61.8534	42	54.878
34	62.0544	11	55.3143
30	62.1275	29	55.7446
22	62.7178	30	57.1157
38	62.8529	43	57.2968
41	65.424	20	58.327
40	66.7514	39	58.7072
43	66.7675	37	60.2237
42	68.9854	19	63.7606
44	74.4596	23	65.2258
		47	67.1832
		48	67.7108
		46	68.2031
		44	68.3094

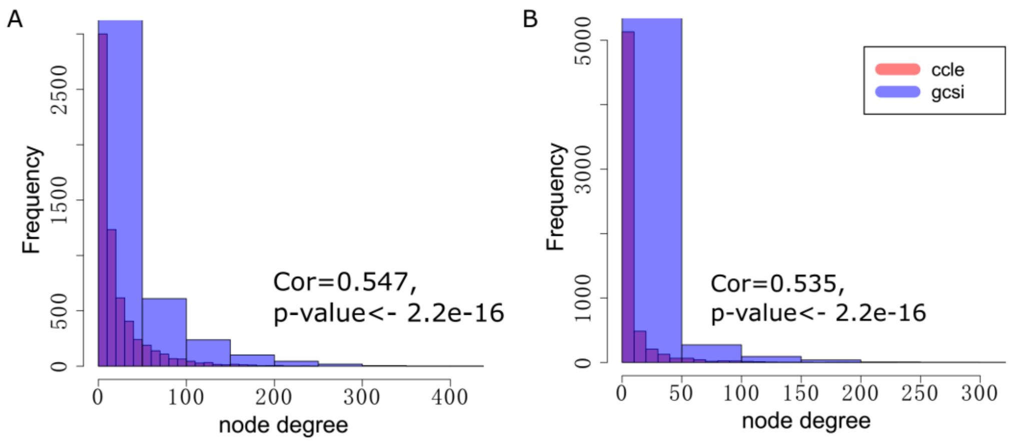

Figure S1. Stability of the networks through the Pearson correlation coefficients (Cor) of each isoform degree in the two networks. A Breast cancer type isoform coexpression network. B I

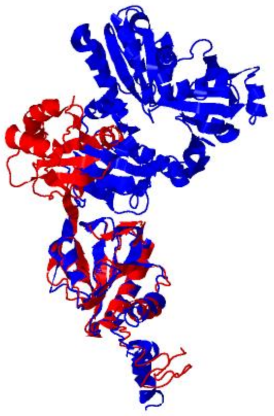

Figure S2. The 3D structures difference of major target isoform and principal isoform of P4HB gene. Principal isoform (ENSP00000327801) with 508 residues in blue, major target isoform (ENSP00000388117) with 274 residues in red.
